# Characterization and transmission of plasmid-mediated multidrug resistance in foodborne *Vibrio parahaemolyticus*

**DOI:** 10.3389/fmicb.2024.1437660

**Published:** 2024-07-31

**Authors:** Haibo Zhou, Zhaoxin Lu, Xinmei Liu, Xiaomei Bie, Xinping Cui, Zuwei Wang, Xiaojie Sun, Jun Yang

**Affiliations:** ^1^College of Food Science and Technology, Nanjing Agricultural University, Nanjing, China; ^2^Key Laboratory of Detection and Traceability Technology of Foodborne Pathogenic Bacteria for Jiangsu Province Market Regulation, Nanjing Institute for Food and Drug Control, Nanjing, China

**Keywords:** *Vibrio parahaemolyticus*, multidrug resistance, whole genome sequencing, plasmid, antimicrobial resistance genes, horizontal transfer

## Abstract

**Objectives:**

The purpose of this study was to determine the structural features and transferability of the multidrug-resistance (MDR) plasmid, and resistance phenotypes for the tested antimicrobials in foodborne *Vibrio parahaemolyticus*.

**Methods:**

Plasmids were isolated from a *V. parahaemolyticus* strain of seafood origin, then sequenced using the Illumina NovaSeq 6000 and PacBio Sequel II sequencing platforms to obtain the complete genome data. Characterization of the MDR plasmid pVP52-1, including determination of antimicrobial resistance genes (ARGs), plasmid incompatibility groups, and transferability, was carried out.

**Results:**

*V. parahaemolyticus* strain NJIFDCVp52 contained two circular chromosomes and two circular plasmids (pVP52-1 and pVP52-2). Plasmid typing indicated that pVP52-1 belonged to the incompatibility group IncA/C_2_ and the sequence type pST3. pVP52-1 carried 12 different ARGs, an IS110-composite transposon consisting of *aac(6′)-Ib*-cr, *qnrVC1*, *aac(6′)-Ib*, *dfrA14*, and the IS26-*mphA*-IS6100 unit flanked by inverted sequences of IS5075 and IS4321. pVP52-2 carried no ARGs. A plasmid elimination assay showed that only pVP52-1 and its ARGs were lost, the loss of resistance to several antimicrobials, causing a change from the ampicillin-ampicillin/sulbactam-cefazolin-cefoxitin-ceftazidime-cefotaxime-imipenem-trimethoprim/sulfamethoxazole resistance pattern to the ampicillin resistance pattern. In accordance, a conjugation transfer assay showed that only pVP52-1 and its ARGs were horizontally transferred, leading to increased antimicrobial resistance in *Escherichia coli* strain EC600, causing a change from the ampicillin-nalidixic acid resistance pattern to the ampicillin-ampicillin/sulbactam-cefazolin-cefoxitin-ceftazidime-cefotaxime-imipenem-nalidixic acid-chloramphenicol-tetracycline-trimethoprim/sulfamethoxazole-azithromycin resistance pattern. Further transferability experiments revealed that pVP52-1 could be transferred to other enterobacterial strains of *E. coli* and *Salmonella*.

**Discussion:**

This study emphasizes the urgent need for continued surveillance of resistance plasmids and changes in antimicrobial resistance profiles among the *V. parahaemolyticus* population.

## 1 Introduction

*Vibrio parahaemolyticus* is one of the most important zoonotic pathogens and is mainly associated with the consumption of raw or undercooked contaminated aquatic products (Campbell et al., 2022; Yang et al., 2022). Antimicrobial agents are widely used for the prevention and treatment of bacterial infections in the fields of agriculture, aquaculture, and clinical medicine, and their main mechanisms are as follows: (I) inhibition of cell wall synthesis (such as *β*-lactams); (II) inhibition of protein synthesis (such as phenicols, tetracyclines, aminoglycosides, and macrolides); (III) inhibition of nucleic acid synthesis (such as quinolones); and (IV) inhibition of folate synthesis (such as sulfonamides and trimethoprim) (Zou et al., 2017). The misuse and overuse of antimicrobials have facilitated the rapid development and spread of antimicrobial resistance (Heffernan, 2022; Sagar et al., 2023). Bacterial infections caused by multidrug-resistant organisms are often responsible for increased morbidity, mortality, and medical costs. Globally, an estimated 4.95 million deaths were associated with antimicrobial resistance in 2019, of which 1.27 million deaths were directly attributable to antimicrobial resistance (Murray et al., 2022). The review on antimicrobial resistance by O'Neill (2014, 2016) reported that antimicrobial resistance could cause 10 million deaths every year by 2050.

The dissemination of antimicrobial resistance is largely attributed to the transfer of antimicrobial resistance genes (ARGs) among different bacterial hosts, including by vertical gene transfer (VGT) and horizontal gene transfer (HGT) (Li et al., 2019). HGT is the dominant mechanism for the transmission of ARGs, and consists of the following three primary pathways: conjugation, transformation, and transduction, with conjugation being the most important route (Li et al., 2021; Zhang et al., 2021). In general, these ARGs are located on mobile genetic elements (MGEs), such as plasmids, insertion sequences (ISs), transposons (Tns), and integrons (Ins) (Partridge et al., 2018; Zhang et al., 2019). Plasmids are extrachromosomal DNA molecules that can be gained and lost within and between bacterial populations, thereby playing a significant role in HGT (Ares-Arroyo et al., 2023). Additionally, it was discovered that plasmids of incompatibility groups IncF, IncI, IncA/C, IncH, IncN, and IncX were considered major carriers of ARGs and conferred a multidrug-resistance (MDR) phenotype, resulting in the widespread dispersion of many classes of ARGs, including *β*-lactamases (*bla*_TEM_, *bla*_CTX-M_, *bla*_CMY_, and *bla*_OXA_), quinolone resistance genes (PMQR), colistin resistance genes (*mcr-1*), enzyme-modifying genes of aminoglycosides (*aadA*, *aphA1*, *aacC*, *strA*, and *strB*), sulfonamides (*sul1*, *sul2*), and trimethoprim (*dfrA*), among others (Rozwandowicz et al., 2018).

At present, there are many reports on different types of resistance plasmids and their horizontal transmission in *Enterobacteriaceae* (Sun et al., 2019; Stosic et al., 2021; Lefèvre et al., 2023; Supa-amornkul et al., 2023) and *Vibrio* spp. (e.g., *Vibrio cholerae* and *Vibrio alginolyticus*) (Wang et al., 2018; Zheng et al., 2018), but there are still few studies on *V. parahaemolyticus* (Liu et al., 2013). In this study, we identified a conjugative plasmid recovered from a *V. parahaemolyticus* strain of seafood origin that carried 12 different ARGs. The objective of this study was to determine the structural features and transferability of the plasmid, and MDR phenotypes for the tested antimicrobials.

## 2 Materials and methods

### 2.1 Bacterial strains

The *V. parahaemolyticus* strain NJIFDCVp52 (sequence type ST1543) was isolated from a local seafood market (Nanjing, China) in 2021 (Zhou et al., 2022). The *Escherichia coli* strain EC600 and all isolates (Supplementary Table S1) used in this study were preserved by the Microbiology Laboratory, Nanjing Institute for Food and Drug Control. Species identification was confirmed by matrix-assisted laser desorption/ionization time of flight mass spectrometry (MALDI-TOF MS, Bruker Daltonics, United States), VITEK-2 system (BioMérieux, France), and strains were stored at −80°C.

### 2.2 Whole genome sequencing

After the strain activation period, a single colony with typical characteristics on Vibrio chromogenic medium (CHROMagar, France) was inoculated in 3% (w/v) NaCl alkaline peptone water (3% NaCl APW; Beijing Land Bridge, China) and cultured at 37°C for 16 h to the late logarithmic growth stage. Genomic DNA was extracted from the cultures using the E.Z.N.A® Bacterial DNA Kit (OMEGA, United States), and the sample was then tested for purity, concentration, and integrity. High-quality DNA (OD260/280 = 1.8–2.0, >5 μg) was considered suitable for subsequent use. Illumina libraries were prepared using the TruSeq™ Nano DNA Sample Prep Kit (Illumina, United States) followed by next-generation sequencing based on the Illumina NovasSeq 6000 platform (Tsingke Biotechnology, Beijing, China) with 2 × 150 bp paired-end reads. Meanwhile, single-molecule real-time (SMRT) sequencing with zero-mode waveguides (ZMWs) was carried out on the PacBio Sequel II platform (Tsingke Biotechnology).

### 2.3 Bioinformatic analysis

Raw data were filtered to obtain clean reads using Trimmomatic v0.36 (Bolger et al., 2014). Both long reads (Sequel II) and short reads (Novaseq 6000) were *de novo* assembled using Unicycler v0.4.8 (Wick et al., 2017) with a hybrid assembly strategy. The circular visualization of assembled genomes was generated using the R package circlize v3.6.3 (Gu et al., 2014). The coding sequences (CDSs) were predicted using GeneMarkS v4.17 (Besemer et al., 2001). Meanwhile, the tRNA, rRNA, and other ncRNA genes were identified by tRNAscan-SE v2.0.4 (Schattner et al., 2005), RNAmmer v1.2 (Lagesen et al., 2007), and Infernal v1.1 (Nawrocki & Eddy, 2013), respectively. The protein sequence of each predicted gene was functionally annotated against multiple databases, including NR (Buchfink et al., 2015), COG (Tatusov et al., 2001), KEGG (Aramaki et al., 2020), GO (Huerta-Cepas et al., 2019), Swissport (Buchfink et al., 2015), CAZy (Zhang et al., 2018), CARD (Altschul et al., 2005), and VFDB (Buchfink et al., 2015). Plasmid replicon types and plasmid multilocus sequence types were determined by PlasmidFinder 2.0 and pMLST (Carattoli et al., 2014). Comparative genomic analysis and generation of plasmid maps were performed using BLAST Ring Image Generator (BRIG) v0.95 (Alikhan et al., 2011) and Easyfig_win_2.2.5 (Sullivan et al., 2011). For structurally variable regions, composite transposon-type structures were observed by the MobileElementFinder database v1.0.2 (Johansson et al., 2021).

### 2.4 Detection of ARGs

PCR assays were used to screen for the presence of *β*-lactamases (*bla*_CARB-4_, *bla*_CARB-18_), quinolones [*qnrVC1*, *aac(6′)-Ib*-cr], aminoglycosides [*aac(6′)-Ib*, *aph(6)-Id*, *aph(3″)-Ib*], phenicol (*floR*), macrolide (*mphA*), tetracyclines (*tet34*, *tet35*, *tetA*), folate synthesis inhibitors (*sul2*, *dfrA1*, *dfrA14*), and the plasmid-specific gene (*vp52004910*). All primers used in this study were synthesized by GenScript Biotech Co., Ltd. (Nanjing, China) and are listed in Table 1. PCR amplification was performed separately in a total volume of 25 μL containing 12.5 μL 2 × SanTaq PCR Mix (with blue dye) (Sangon Biotech, Shanghai, China), 400 nM of each primer, 1 μL of DNA template, and sterile distilled water to adjust the volume. Amplification reactions were carried out in a C1000 Touch™ Thermal Cycler (Bio-Rad, United States) using the following thermal conditions: initial denaturation at 94°C for 5 min, followed by 35 cycles of denaturation at 94°C for 30 s, annealing at 59°C for 30 s, extension at 72°C for 20 s, and a final extension at 72°C for 10 min. 5 μL of the PCR amplicons were confirmed by agarose gel (Biowest, Spain) electrophoresis on a 2% (w/v) gel mixed with GeneGreen nucleic acid dye (Tiangen Biotech, Beijing, China) and visualized by a GelDoc XR + Gel imaging system (Bio-Rad, United States).

**Table 1:** Primers used in this study.

Target gene	Forward primer (5′–3′)	Reverse primer (5′–3′)	Product size (bp)
*bla*_CARB-4_	ATGCAGCAGCAAATATCATCC	TGTCGTATCCCTCAAATCACC	149
*bla*_CARB-18_	ATTGGAGTGTCAGTCTGGGATA	GTCGCTTAGCATGGTGGC	117
*qnrVC1*	AAATTGCTTTGGTATTGAACTGA	GCTCAAAATTGGCATAGGATAAG	146
*aac(6′)-Ib-*cr	CGCCCGACACTTGCTGAC	ATCGGTTTCTTCTTCCCACCT	162
*aph(3″)-Ib*	AAATCGCACCTGCTTCCC	GCACCCTCCTGTTCCTCC	121
*aph(6)-Id*	ACCTTTTCCAGCCTCGTTT	GACTGCTCCCCTCCCATT	141
*aac(6′)-Ib*	CGCCCGACACTTGCTGAC	ATCGGTTTCTTCTTCCCACCA	162
*floR*	CTCGCCGTCATTCCTCAC	CGATGTCGTCGAACTCTGC	110
*mphA*	GTTCGTCCACGCCCGACT	GAGCATCCCCGCATCCAC	122
*tet34*	GGAAAGGTATTTGGGCGGTAA	GATCATGGTCGTAGCTTGAGAT	114
*tet35*	TCTAATGGCGTTCTCAACCG	CCAAAACCGCACTTAGCATA	122
*tetA*	CAGGCAGGTGGATGAGGAA	GCATAGATCGCCGTGAAGAG	105
*sul2*	TTTCGGCATCGTCAACATAA	CGTCGGGATTGCTGGAT	146
*dfrA1*	ACCCAACCGAAAGTATGCG	CCACCACCTGAAACAATGACA	138
*dfrA14*	AACGGCGTGATTGGTTGC	CGTATTTCCTATTGGGGAGTGC	145
*vp52004910*	CCACATAAAGGTCAATATCCTCG	AAAAGTCCTCCAACTCAGCAA	105

### 2.5 Antimicrobial susceptibility testing

The antimicrobial resistance profiles were determined by the Kirby-Bauer disk diffusion method and interpreted according to the Clinical and Laboratory Standards Institute (CLSI) breakpoints (CLSI, 2016). The 15 antimicrobial disks belonging to nine different classes (Hangzhou Microbial Reagent, China) included penicillins and *β*-lactam/*β*-lactamase inhibitor combinations (ampicillin, AMP; ampicillin/sulbactam, SAM), cephalosporins (cefazolin, CFZ; cefoxitin, FOX; ceftazidime, CAZ; cefotaxime, CTX), carbapenems (imipenem, IPM), quinolones and fluoroquinolones (nalidixic acid, NAL; ciprofloxacin, CIP; ofloxacin, OFX), phenicols (chloramphenicol, CHL), tetracyclines (tetracycline, TET), aminoglycosides (gentamicin, GEN), folate synthesis inhibitors (trimethoprim/sulfamethoxazole, SXT), and macrolides (azithromycin, AZI). The detailed experimental steps were performed with reference to the method described previously (Zhou et al., 2022). *Escherichia coli* ATCC 25922 was used as a quality control strain.

### 2.6 Plasmid elimination assay

A plasmid elimination assay was performed by sodium dodecyl sulfate (SDS) and a high-temperature culture method as published previously (Letchumanan et al., 2015) with slight modifications. Briefly, a single colony on Vibrio chromogenic medium was picked and used to inoculate 3% NaCl tryptone soy broth (TSB, Beijing Land Bridge) containing 0.025% SDS (w/v) (Solarbio, Beijing, China); the culture was incubated at 43°C for 16 h with shaking. Then, 200 μL of culture was used to inoculate 10 mL of 3% NaCl TSB medium (SDS free), and the culture was incubated at 43°C for 16 h with shaking. Finally, 200 μL of culture was used to inoculate 10 mL of 3% NaCl TSB medium (SDS free), and the culture was incubated at 37°C for 16 h with shaking. Plasmid DNA was subsequently extracted using the FastPure® Plasmid Mini Kit (Vazyme, Nanjing, China) following the manufacturer’s instructions. Plasmid elimination strains were confirmed by antimicrobial susceptibility testing and PCR detection of resistance genes. In this study, the plasmid of the *V. parahaemolyticus* NJIFDCVp52 could be effectively eliminated by the above procedure. If the plasmid could not be successfully eliminated in the first round of trials, the elimination steps were repeated multiple times. If necessary, this was achieved by gradually increasing the concentration of SDS (0.03–0.4%).

### 2.7 Conjugation transfer assay

To evaluate the transferability of the MDR plasmid in the *V. parahaemolyticus* NJIFDCVp52, conjugation transfer experiments were carried out using the broth mating method as described previously (Couturier et al., 2023) with minor modifications. A rifampicin-resistant *E. coli* EC600 strain was used as the recipient. In brief, both donor and recipient strains were cultured to the logarithmic growth stage in lysogeny broth (LB: 1% tryptone, 0.5% yeast extract, 1% NaCl; Beijing Land Bridge). Overnight cultures were streaked onto Eosin-Methylene blue agar (EMB, Beijing Land Bridge) medium supplemented with either 4 μg/mL tetracycline (TET; Aladdin, Shanghai, China), 64 μg/mL rifampicin (RIF, Aladdin), or both antimicrobials (4 μg/mL TET and 64 μg/mL RIF). The cultures were then incubated at 37°C for 24–48 h and checked for the microbial growth. Meanwhile, independent overnight cultures were collected by centrifugation and resuspended to a 0.5 McFarland turbidity standard in 0.85% (w/v) sterilized NaCl solution (sterile saline). 200 μL of the donor strain (*V. parahaemolyticus* NJIFDCVp52) and 200 μL of the recipient strain (*E. coli* EC600) were thoroughly mixed at a ratio of 1:1 and statically incubated at 37°C for 4 h. The conjugation mixture was then diluted with sterile saline solution and uniformly plated onto EMB plates containing 4 μg/mL TET and 64 μg/mL RIF. Subsequently, donor and recipient strains were also subjected separately to the same operation as quality control. Putative transconjugants were determined with antimicrobial susceptibility testing and PCR detection of resistance genes. The identified transconjugants were kept in drug-containing medium with sterile 20% (v/v) glycerol and stored at −80°C.

To further investigate the transferability of plasmid pVP52-1 from the *V. parahaemolyticus* NJIFDCVp52 to other bacterial species, conjugation transfer experiments were also carried out for *E. coli* (20 isolates), *Bacillus cereus* (20 isolates), *Pseudomonas aeruginosa* (nine isolates), *Listeria monocytogenes* (seven isolates), *Salmonella* (20 isolates) (including 8 *S. Indiana*, 2 *S*. Chester, 7 *S. typhimurium*, and 3 *S*. Derby), and *Staphylococcus aureus* (20 isolates). Conjugation transfer was conducted using the same experimental steps as described above. Details of isolates, drugs, and culture media were listed in Supplementary Table S1.

### 2.8 Conjugation transfer frequency and plasmid stability

Conjugation transfer frequency was examined using the conventional plate count method. The previous steps were the same as the described method in Section 2.7. The frequency was calculated as the number of transconjugants (EMB plates containing 4 μg/mL TET and 64 μg/mL RIF) divided by the number of recipient cells (EMB plates containing 64 μg/mL RIF) (Chen et al., 2020). The values are expressed as the mean ± standard deviation of three independent experiments. To assess plasmid stability, the plasmid loss of transconjugants was validated by subculturing in antimicrobial-free medium. A single colony on the double-selective medium (EMB plates containing 4 μg/mL TET and 64 μg/mL RIF) was inoculated into antimicrobial-free LB medium and cultured at 37°C for 24 h, followed by 24 h growth of 15 consecutive subcultures at 1% inoculation amount, with sampling and plating every three subcultures. The cultures were diluted with sterile saline solution (10^−7^ and 10^−8^ dilution factors) and uniformly plated onto antimicrobial-free EMB plates. Fifty single colonies were randomly selected from an appropriate dilution for further PCR verification (resistance gene *tetA*), three parallels for each dilution.

### 2.9 Nucleotide sequence accession numbers

The complete sequences of *V. parahaemolyticus* NJIFDCVp52 containing two chromosomes and two plasmids have been deposited in the GenBank database under accession numbers CP128803-CP128806.

## 3 Results

### 3.1 Complete sequences of multidrug-resistant strain

The genomic DNA extracted from *V. parahaemolyticus* strain NJIFDCVp52 was sequenced using the Illumina NovaSeq 6000 and PacBio Sequel II sequencing platforms. The polished genome assembly for NJIFDCVp52 included two circular chromosomes of 3,398,295 bp (Chr1) and 1,742,377 bp (Chr2), along with two circular plasmids of 172,213 bp (Plasmid1) and 85,030 bp (Plasmid2). In total, 5,038 annotated CDSs were identified with 4,678 CDSs in the chromosomes and 360 CDSs in the plasmids, accounting for 86.3% of the total genome length. The circle diagram clearly displays the characteristics of the two chromosomes and two plasmids (Supplementary Figure S1).

Two circular plasmids were identified in the *V. parahaemolyticus* NJIFDCVp52, named pVP52-1 (172,213 bp) and pVP52-2 (85,030 bp). pVP52-1 was an incompatibility group IncA/C_2_ plasmid and belonged to pST3 (profile: *repA*-2, *parA*-2, *parB*-2, and *A053*-1); pVP52-2 was an untypeable plasmid. Furthermore, a total of 15 ARGs related to seven types of antimicrobials were recognized, 12 [*tetA*, *sul2*, *dfrA1*, *dfrA14*, *bla*_CARB-4_, *qnrVC1*, *aac(6′)-Ib*, *aac(6′)-Ib*-cr, *aph(6)-Id*, *aph(3″)-Ib*, *floR*, *mphA*] of which were located on pVP52-1, two (*tet34*, *tet35*) of which were located on Chr1 and *bla*_CARB-18_ was located on Chr2, while no other ARGs were found on pVP52-2.

### 3.2 Characteristics of plasmid pVP52-1

**Figure 1:**
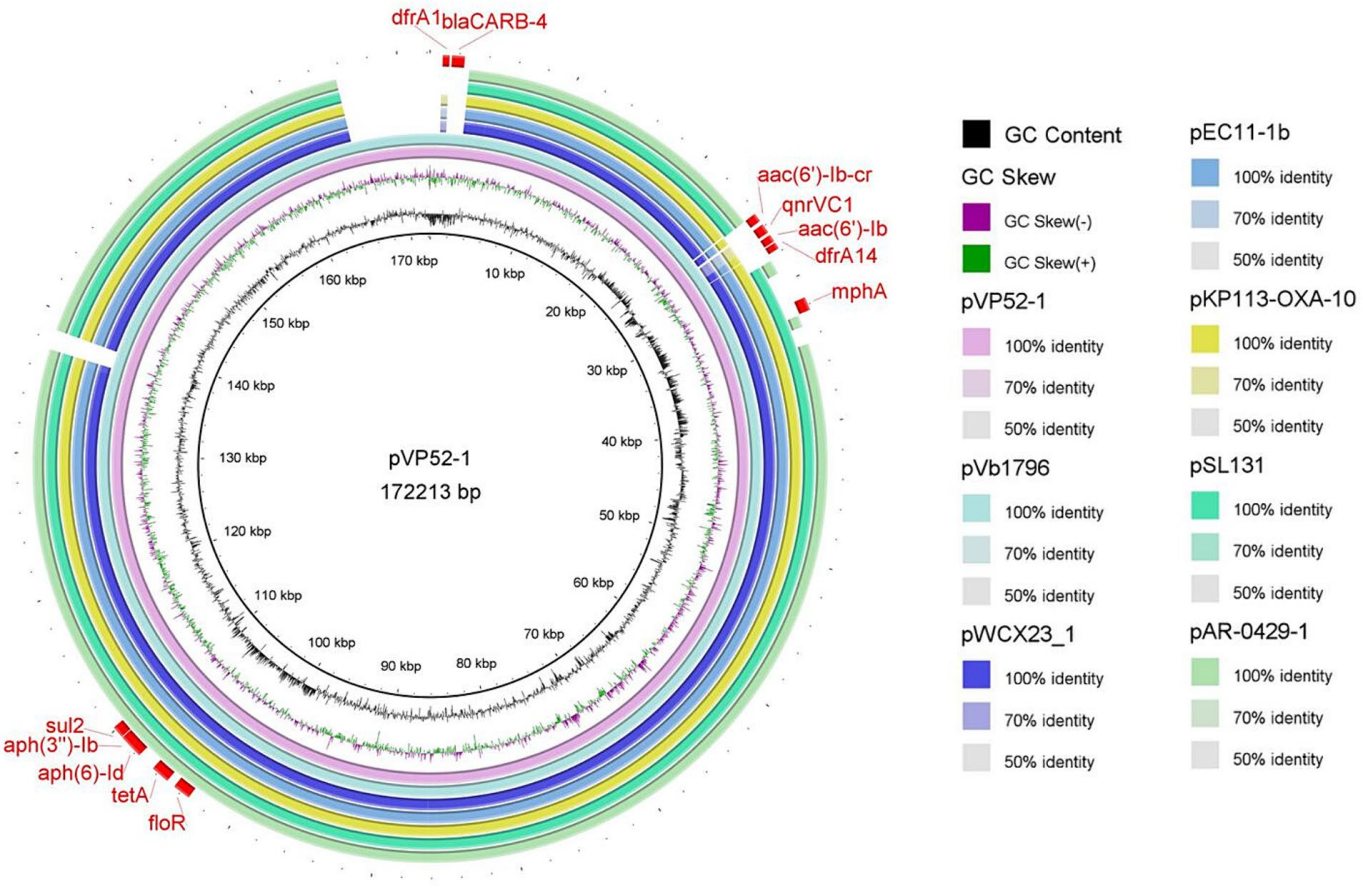
Circle comparisons of the whole pVP52-1 sequence with similar plasmids. From the inside outwards: the innermost circle represents the genome size; the second and third circles represent GC content and GC skew; the fourth circle represents pVP52-1 from *Vibrio parahaemolyticus* strain NJIFDCVp52; the fifth circle represents pVb1796 from *Vibrio alginolyticus* strain; the sixth circle represents pWCX23_1 from *Aeromonas hydrophila* strain; the seventh circle represents pEC11-1b from *Escherichia coli* strain; the eighth circle represents KP113-OXA-10 from *Klebsiella pneumoniae* strain; the ninth circle represents pSL131 from *Salmonella enterica* subsp. *enterica* strain; the tenth circle represents pAR-0429-1 from *Escherichia coli* O157 strain; and the outermost circle represents ARGs of the reference sequence pVP52-1. Visualization of the BLAST analysis and ARGs of annotation was performed with BRIG v0.95.

The full sequence of pVP52-1 was aligned by nucleotide BLAST against the NCBI database. As shown in Figure 1, the comparison results showed that pVP52-1 exhibited high homology with *V. alginolyticus* plasmid pVb1796 (accession number: MH113855.1; 100% coverage, 99.99% identity), *Aeromonas hydrophila* plasmid pWCX23_1 (accession number: CP028419.1; 93% coverage, 99.99% identity), *E. coli* plasmid pEC11-1b (accession number: MT559994.1; 93% coverage, 99.92% identity), *Klebsiella pneumoniae* plasmid KP113-OXA-10 (accession number: ON023483.1; 93% coverage, 99.92% identity), *Salmonella enterica* subsp. *enterica* plasmid pSL131 (accession number: MH105050.1; 91% coverage, 99.90% identity), and *E. coli* O157 plasmid pAR-0429-1 (accession number: CP044142.1; 89% coverage, 99.96% identity).

**Figure 2:**
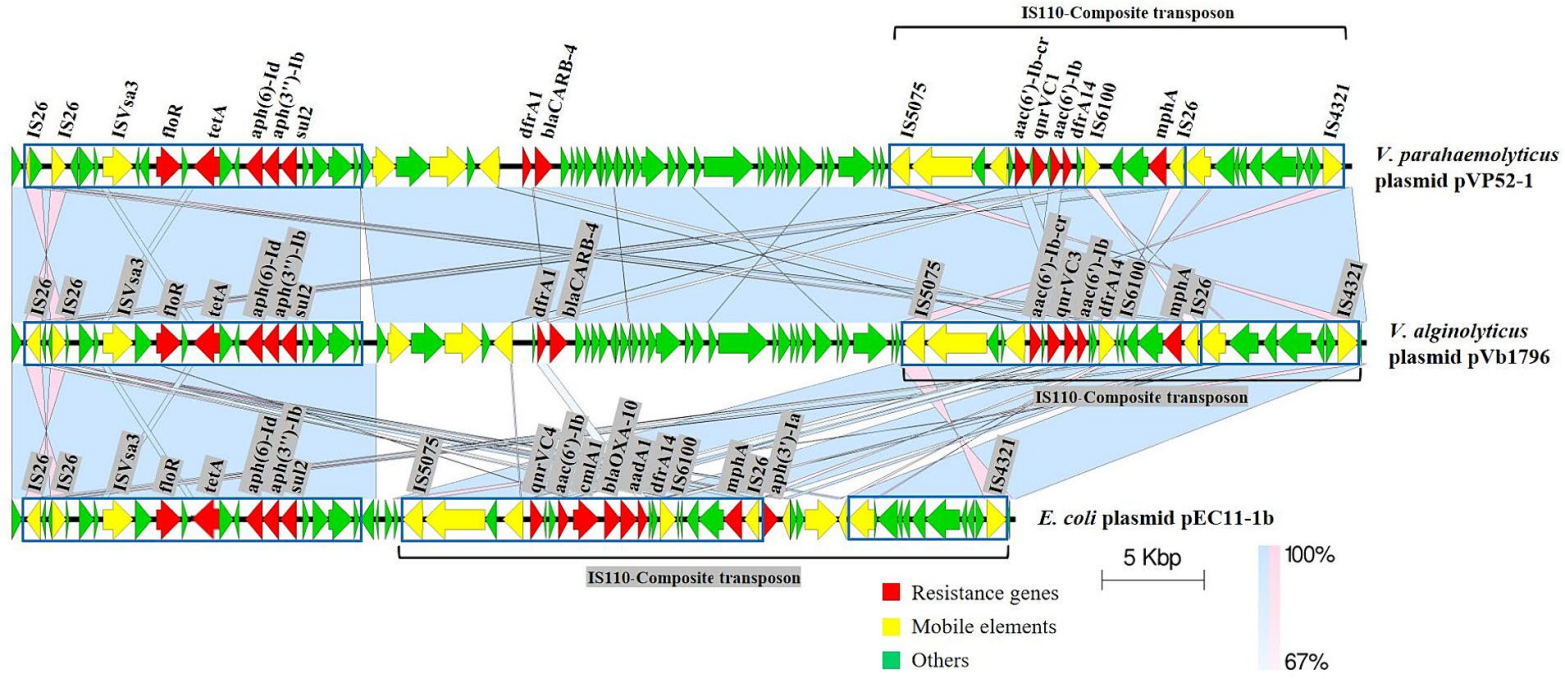
Linear comparisons of pVP52-1 resistance regions with similar plasmids. Arrows represent the positions and transcriptional directions of genes, and colors represent gene functions. Shaded areas indicate positive genetic similarity (blue) and reverse genetic similarity (pink) in the corresponding region between plasmids. The alignment was performed with Easyfig_win_2.2.5.

Specifically, the IS110-composite transposon (22,778 bp) containing *aac(6′)-Ib*-cr, *qnrVC1*, *aac(6′)-Ib*, *dfrA14* and the IS26-*mphA*-IS6100 unit was flanked by inverted sequences of IS5075 and IS4321. The resistance gene cluster *floR*-*tetA*-*aph(6)-Id*-*aph(3″)-Ib*-*sul2* had an upstream insertion sequence ISVsa3, and *dfrA1*-*bla*_CARB-4_ also had several MGEs (Figure 2). pVb1796 from *Vibrio alginolyticus* strain Vb1796 carried the same ARG pattern [*tetA*, *sul2*, *dfrA1*, *dfrA14*, *bla*_CARB-4_, *qnrVC1*, *aac(6′)-Ib*, *aac(6′)-Ib*-cr, *aph(6)-Id*, *aph(3″)-Ib*, *floR*, *mphA*] as pVP52-1. Furthermore, the genetic environment of ARGs between the two plasmids was basically consistent. pEC11-1b from *E. coli* strain EC11 also contained a composite transposon (IS110-composite transposon, 29,626 bp) (Zhang et al., 2020). The main difference was that the IS110-composite transposon of pEC11-1b harbored different types of ARGs [*qnrVC4*, *aac(6′)-Ib*, *cmlA1*, *bla*_OXA-10_, *aadA1*, *dfrA14*, *aph(3′)-Ia*], which shared an identical core structure with pVP52-1. Compared with pEC11-1b, there were some additional MGEs, ARGs (*dfrA1*, *bla*_CARB-4_) and hypothetical proteins between the two highly similar regions on pVP52-1.

### 3.3 Plasmid elimination assay

**Figure 3:**
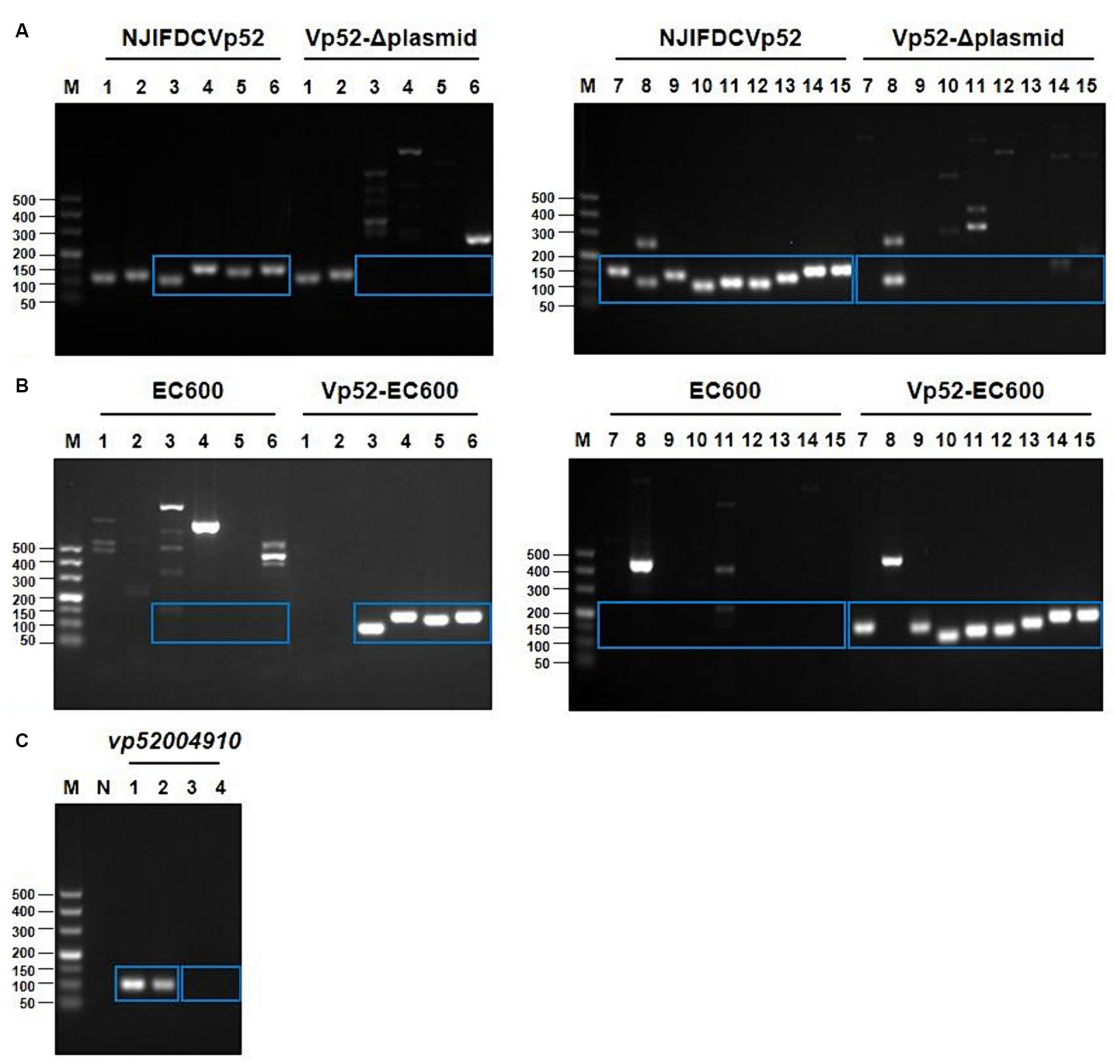
PCR amplification products of ARGs and the plasmid-specific gene. 15 different ARGs were detected by PCR before and after plasmid elimination **(A)** and conjugation transfer **(B)**. pVP52-2-mediated specific gene (*vp52004910*) was detected by PCR before and after plasmid elimination **(C)**. **(A,B)** M, DL500 DNA marker; Lane 1, *tet34*; Lane 2, *tet35*; Lane 3, *tetA*; Lane 4, *sul2*; Lane 5, *dfrA1*; Lane 6, *dfrA14*; Lane 7, *bla*_CARB-4_; Lane 8, *bla*_CARB-18_; Lane 9, *qnrVC1*; Lane 10, *floR*; Lane 11, *mphA*; Lane 12, *aph(3″)-Ib*; Lane 13, *aph(6)-Id*; Lane 14, *aac(6′)-Ib*-cr; Lane 15, *aac(6′)-Ib*. **(C)** M, DL500 DNA marker; N, blank control; Lane 1, NJIFDCVp52; Lane 2, Vp52-Δplasmid; Lane 3, EC600; Lane 4, Vp52-EC600. The blue rectangle indicate the area of target bands.

Based on the whole genome sequencing data of *V. parahaemolyticus* strain NJIFDCVp52, plasmid-eliminated strains were screened using PCR for the presence of 15 ARGs. Electrophoresis results showed that the wild-type strain NJIFDCVp52 carried all 15 ARGs (Figure 3A). The plasmid elimination strain named Vp52-Δplasmid was successfully obtained in the plasmid elimination experiment. From Figure 3A, the 12 ARGs [*tetA*, *sul2*, *dfrA1*, *dfrA14*, *bla*_CARB-4_, *qnrVC1*, *aac(6′)-Ib*, *aac(6′)-Ib*-cr, *aph(6)-Id*, *aph(3″)-Ib*, *floR*, *mphA*] were not detected in the strain Vp52-Δplasmid; however, the *tet34*, *tet35*, and *bla*_CARB-18_ genes were still detected. These results indicated that the 12 ARGs were located on the plasmid and lost together with plasmid elimination, and the *tet34*, *tet35*, and *bla*_CARB-18_ genes were localized chromosomally, which was congruent with the sequencing results. To further validate the number of plasmids removed, a DNA fragment of approximately 105 bp from pVP52-2 was amplified by using specific primers for the *vp52004910* gene. As shown in Figure 3C, the *vp52004910* gene was present in both the *V. parahaemolyticus* NJIFDCVp52 and Vp52-Δplasmid strains. Thus, pVP52-1 carrying 12 ARGs was obviously lost, and pVP52-2 carrying no ARGs was not lost during plasmid elimination.

### 3.4 Plasmid transfer from NJIFDCVp52 to EC600

#### 3.4.1 Conjugation transfer assay

The conjugation transfer showed that the *V. parahaemolyticus* NJIFDCVp52 plasmid could be successfully transferred to an *E. coli* recipient (strain EC600) with a frequency of 2.9 ± 0.078 × 10^−4^. Similarly, a transconjugant (designated Vp52-EC600) was utilized for the detection of 15 ARGs (Figure 3B). Strain EC600 by itself did not possess any of these 15 ARGs, as no amplified bands were detected by agarose gel electrophoresis. The 12 ARGs carried on pVP52-1 [*tetA*, *sul2*, *dfrA1*, *dfrA14*, *bla*_CARB-4_, *qnrVC1*, *aac(6′)-Ib*, *aac(6′)-Ib*-cr, *aph(6)-Id*, *aph(3″)-Ib*, *floR*, *mphA*] were detected in the *E. coli* Vp52-EC600, while three chromosome-mediated ARGs (*tet34*, *tet35*, and *bla*_CARB-18_) were not detected, as shown in Figure 3B. The results of plasmid stability assay indicated that plasmid loss did not occur during serial generation in antimicrobial-free medium (Table 2). Accordingly, only the plasmid pVP52-1 along with its ARGs could be transferred into the recipient by conjugation.

**Table 2:** Plasmid stability in transconjugants.

Transconjugant	Stability^a^ (%)				
	3 days	6 days	9 days	12 days	15 days
Vp52-EC600	100	100	100	100	100

^a^Data are shown as the proportion of strains maintaining the plasmid at different time points.

#### 3.4.2 Antimicrobial susceptibility analysis

**Figure 4:**
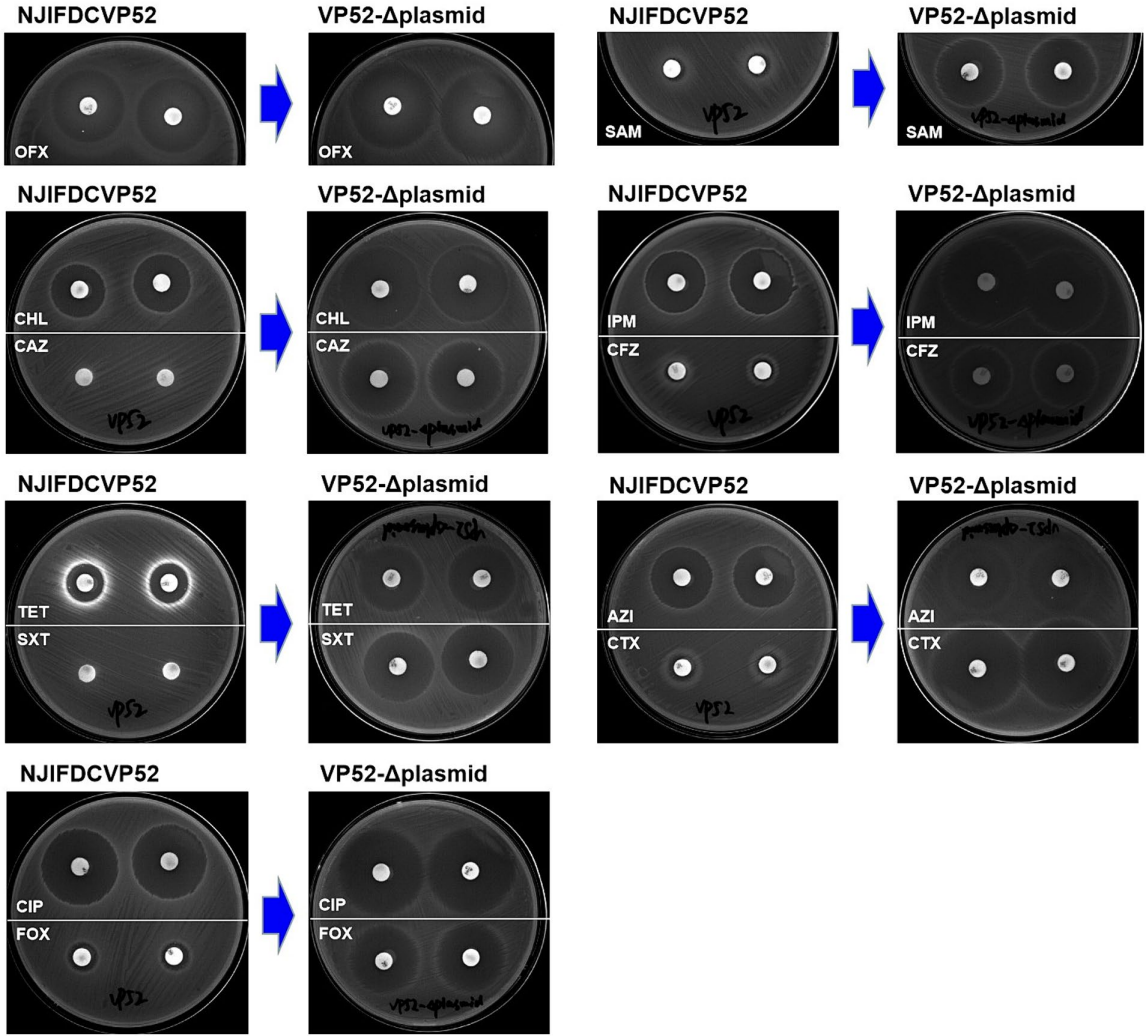
Inhibitory zone of *Vibrio parahaemolyticus* strain NJIFDCVp52 before and after plasmid elimination. OFX, Ofloxacin; CHL, Chloramphenicol; CAZ, Ceftazidime; TET, Tetracycline; SXT, Trimethoprim/sulfamethoxazole; CIP, Ciprofloxacin; FOX, Cefoxitin; SAM, Ampicillin/Sulbactam; IPM, Imipenem; CFZ, Cefazolin; AZI, Azithromycin; and CTX, Cefotaxime.

**Figure 5:**
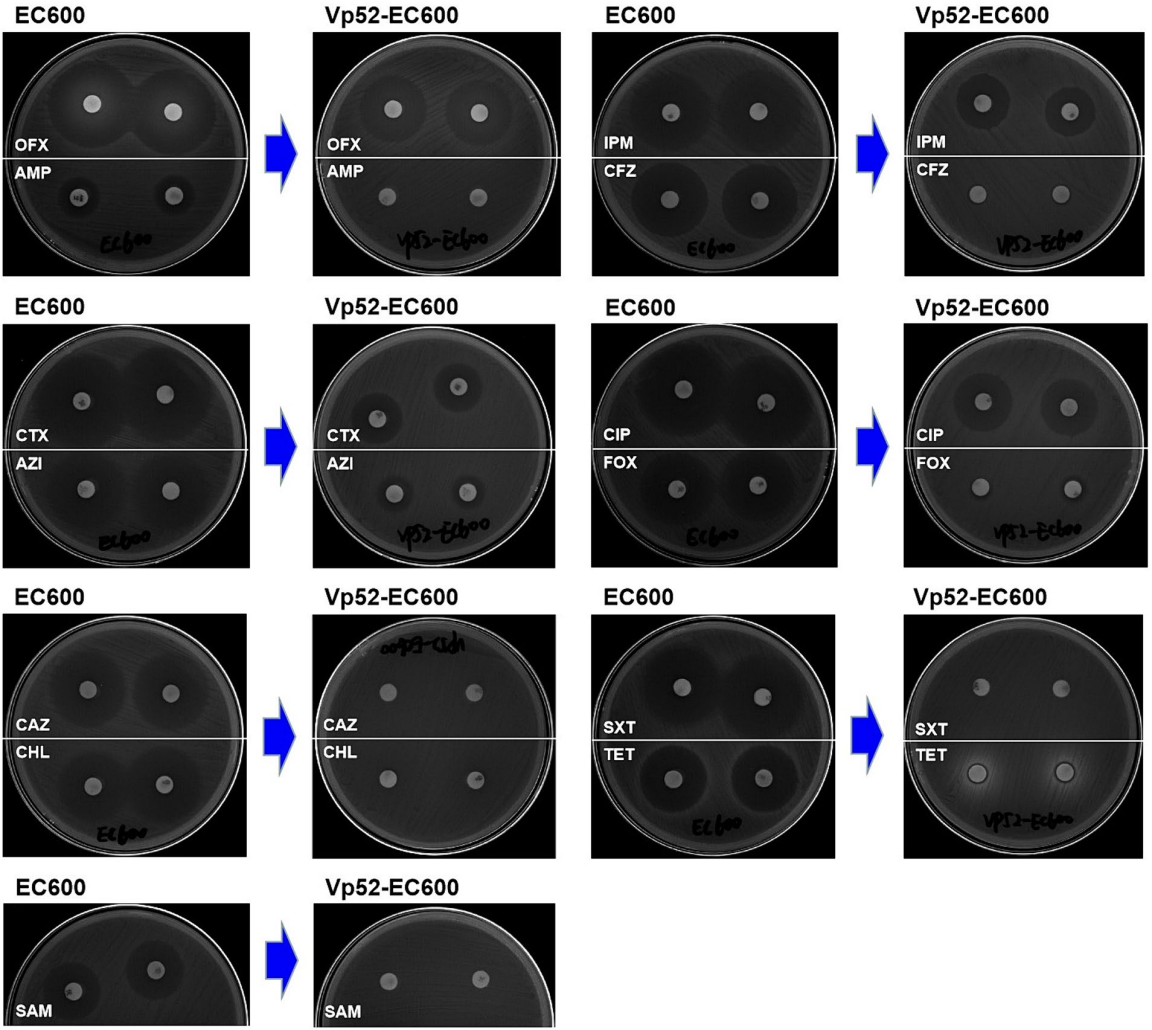
Inhibitory zone of *Escherichia coli* strain EC600 before and after conjugation transfer. OFX, Ofloxacin; AMP, Ampicillin; CTX, Cefotaxime; AZI, Azithromycin; CAZ, Ceftazidime; CHL, Chloramphenicol; SAM, Ampicillin/Sulbactam; IPM, Imipenem; CFZ, Cefazolin; CIP, Ciprofloxacin; FOX, Cefoxitin; SXT, Trimethoprim/Sulfamethoxazole; and TET, Tetracycline.

Susceptibility analysis of 15 antimicrobials was conducted using the disk diffusion method. By comparison, obvious phenotypic differences in the diameters of the inhibition zone were found between the *V. parahaemolyticus* NJIFDCVp52 and Vp52-Δplasmid strains when tested against 12 antimicrobials (except for AMP, NAL, and GEN), including OFX, CHL, CAZ, TET, SXT, CIP, FOX, SAM, IPM, CFZ, AZI, and CTX (Figure 4), which means there was a decrease in antimicrobial resistance from NJIFDCVp52 to Vp52-Δplasmid. In the same way, there were also noticeable differences between the *E. coli* EC600 and Vp52-EC600 strains when tested against 13 antimicrobials (except for NAL and GEN), including AMP, OFX, CHL, CAZ, TET, SXT, CIP, FOX, SAM, IPM, CFZ, AZI, and CTX (Figure 5), which means there was an increase in antimicrobial resistance from EC600 to Vp52-EC600.

#### 3.4.3 Contribution of ARGs to antimicrobial susceptibility

Originally, the *V. parahaemolyticus* NJIFDCVp52 exhibited simultaneous resistance to eight antimicrobials (AMP, SAM, CFZ, FOX, CAZ, CTX, IPM, and SXT). After the elimination of the plasmid (pVP52-1), the strain was sensitive to SAM, FOX, CAZ, CTX, IPM, and SXT but showed intermediate resistance to CFZ and maintained resistance to AMP. That is, the *V. parahaemolyticus* NJIFDCVp52 with the MDR profile AMP-SAM-CFZ-FOX-CAZ-CTX-IPM-SXT was reduced to the *V. parahaemolyticus* Vp52-Δplasmid with the profile AMP (Table 3). In addition, the diameters of the inhibition zone of the Vp52-Δplasmid strain to OFX, CIP, AZI, CHL, and TET were slightly increased, and the resistance phenotypes of OFX, CIP, and AZI remained unchanged below breakpoints; however, that of CHL and TET changed (intermediate resistance to susceptibility).

**Table 3:** Resistance phenotypes of strains before and after plasmid elimination and conjugation transfer.

Antimicrobial agents	Resistance phenotype (diameter/mm)					
	*V. parahaemolyticus* Vp52	*V. parahaemolyticus* Vp52-Δplasmid	Difference	*E. coli* EC600	*E. coli* Vp52-EC600	Difference
Penicillins and β-lactam/β-lactamase inhibitor combinations						
Ampicillin (AMP)	*R* (0)	R (0)	0	R (12.52 ± 0.26)	R (0)	12.52 ± 0.26
Ampicillin/sulbactam (SAM)	*R* (0)	***S*** (22.45 ± 0.84)	22.45 ± 0.84	S (19.47 ± 0.36)	***R*** (0)	19.47 ± 0.36
Cephalosporins						
Cefazolin (CFZ)	*R* (0)	***I*** (20.19 ± 0.34)	20.19 ± 0.34	S (26.90 ± 0.33)	***R*** (0)	26.90 ± 0.33
Cefoxitin (FOX)	*R* (9.38 ± 0.22)	***S*** (20.91 ± 0.18)	11.53 ± 0.39	S (21.21 ± 0.16)	***R*** (0)	21.21 ± 0.16
Ceftazidime (CAZ)	*R* (0)	***S*** (23.90 ± 0.24)	23.90 ± 0.24	S (23.59 ± 0.45)	***R*** (0)	23.59 ± 0.45
Cefotaxime (CTX)	*R* (0)	***S*** (28.87 ± 0.32)	28.87 ± 0.32	S (28.86 ± 0.63)	***R*** (16.73 ± 0.72)	12.14 ± 0.26
Carbapenems						
Imipenem (IPM)	*R* (18.55 ± 0.25)	***S*** (26.64 ± 0.38)	8.09 ± 0.62	S (23.56 ± 0.53)	***R*** (18.51 ± 0.35)	5.05 ± 0.38
Quinolones and fluoroquinolones						
Nalidixic acid (NAL)	*S* (27.84 ± 0.46)	S (28.51 ± 0.50)	0.66 ± 0.04	R (0)	R (0)	0
Ciprofloxacin (CIP)	*S* (22.34 ± 0.20)	S (25.42 ± 0.22)	3.08 ± 0.27	S (27.44 ± 0.31)	***I*** (21.57 ± 0.26)	5.87 ± 0.50
Ofloxacin (OFX)	*S* (22.50 ± 0.13)	S (25.49 ± 0.21)	2.99 ± 0.08	S (29.20 ± 0.65)	S (22.54 ± 0.20)	6.66 ± 0.55
Phenicols						
Chloramphenicol (CHL)	*I* (17.19 ± 0.19)	***S*** (25.80 ± 0.43)	8.61 ± 0.24	S (22.93 ± 0.51)	***R*** (0)	22.93 ± 0.51
Tetracyclines						
Tetracycline (TET)	*I* (13.59 ± 0.30)	***S*** (18.59 ± 0.34)	5.01 ± 0.11	S (22.61 ± 0.38)	***R*** (0)	22.61 ± 0.38
Aminoglycosides						
Gentamicin (GEN)	*S* (18.97 ± 0.47)	S (19.20 ± 0.48)	0.23 ± 0.03	S (22.85 ± 0.46)	S (22.47 ± 0.42)	0.37 ± 0.05
Folate synthesis inhibitors						
Trimethoprim/sulfamethoxazole (SXT)	*R* (0)	***S*** (19.63 ± 0.15)	19.63 ± 0.15	S (26.97 ± 0.59)	***R*** (0)	26.97 ± 0.59
Macrolides						
Azithromycin (AZI)	*S* (18.38 ± 0.26)	S (20.58 ± 0.09)	2.20 ± 0.18	S (21.65 ± 0.25)	***R*** (11.50 ± 035)	10.15 ± 0.19

R, Resistant; I, Intermediate; S, Susceptible. Letters in bold italics indicate changes in the resistance phenotype. Data shown as diameter mean ± standard deviation.

Notably, the conjugation transfer of the MDR plasmid (pVP52-1) facilitated the enhancement of antimicrobial resistance from the *E. coli* strain EC600 with the resistance profile AMP-NAL to the *E. coli* Vp52-EC600 with the MDR profile AMP-SAM-CFZ-FOX-CAZ-CTX-IPM-NAL-CHL-TET-SXT-AZI (Table 3). The diameters of the inhibition zone of Vp52-EC600 to CIP and OFX were slightly reduced, and the level of resistance to CIP was modestly enhanced from susceptibility to intermediate resistance; however, that of OFX remained unchanged below the breakpoint. The characterization of resistance phenotypes and the results of these two assessments (plasmid elimination and conjugation transfer) were mutually consistent.

Combining the results from plasmid elimination and conjugation transfer in this study, *bla*_CARB-4_ was clearly responsible for SAM, CFZ, FOX, CAZ, CTX, and IPM resistance; *floR* was responsible for CHL resistance; *tetA* was responsible for TET resistance; and *sul2*, *dfrA1*, and *dfrA14* were responsible for SXT resistance. With respect to CIP and AZI resistance, although there were apparent differences in the diameters of the resistance zone, resistance phenotypes did not change in the plasmid eliminated strain Vp52-Δplasmid. Nonetheless, resistance phenotypes changed in the transconjugant Vp52-EC600, which could be explained by differences in the genetic backgrounds in these species. The presence of *qnrVC1* [and *aac(6′)-Ib*-cr] and *mphA* was also strongly associated with CIP and AZI resistance, respectively. Moreover, unexpectedly, the presence of *aac(6′)-Ib*, *aac(6′)-Ib*-cr, *aph(6)-Id* and *aph(3″)-Ib* showed no relationship with the GEN resistance.

### 3.5 Plasmid transfer from NJIFDCVp52 to other isolates

Based on preliminary experimental results of antimicrobial susceptibility, selective chromogenic plates containing drugs were used to screen for their corresponding transconjugants (Supplementary Table S1). Among various foodborne pathogenic bacteria, plasmid pVP52-1 could be transferred to the recipient isolates of *E. coli* (transfer frequency, 4.6 ± 0.38 × 10^−5^ ~ 1.4 ± 0.088 × 10^−3^) and *Salmonella* (including *S. Indiana*, *S*. Chester) (transfer frequency, 1.7 ± 0.063 × 10^−5^ ~ 5.4 ± 0.29 × 10^−5^) by conjugation (Table 4). On the contrary, pVP52-1 could not be transferred to the recipient isolates of *B. cereus*, *P. aeruginosa*, *L. monocytogenes*, and *S. aureus*.

**Table 4:** Conjugation transfer frequency of plasmid pVP52-1 from NJIFDCVp52 to *Escherichia coli* and *Salmonella* isolates.

*E. coli* isolate	Transfer frequency^a^	*E. coli* isolate	Transfer frequency^a^	*Salmonella* isolate	Transfer frequency^a^
EC1	2.1 ± 0.16 × 10^−4^	EC27	1.4 ± 0.088 × 10^−3^	*S. Indiana* SC109	1.7 ± 0.063 × 10^−5^
EC3	1.2 ± 0.085 × 10^−4^	EC28	7.8 ± 0.27 × 10^−5^	*S. Chester* SC111	3.9 ± 0.20 × 10^−5^
EC4	4.6 ± 0.38 × 10^−5^	EC29	5.8 ± 0.32 × 10^−4^	*S. Indiana* SC116	2.6 ± 0.075 × 10^−5^
EC6	1.3 ± 0.075 × 10^−4^	EC32	2.0 ± 0.18 × 10^−4^	*S. Indiana* SC124	2.1 ± 0.16 × 10^−5^
EC9	6.1 ± 0.51 × 10^−5^	EC33	5.1 ± 0.42 × 10^−4^	*S. Indiana* SC157	5.4 ± 0.29 × 10^−5^
EC26	7.3 ± 0.67 × 10^−4^	EC34	2.5 ± 0.17 × 10^−4^	*S. Indiana* SC173	5.0 ± 0.22 × 10^−5^

^a^Data are shown as mean ± standard deviation.

## 4 Discussion

*Vibrio parahaemolyticus* is a common foodborne pathogen with high survival and incidence rates worldwide, predominantly causing gastroenteritis and diarrhea (Baker-Austin et al., 2018; Fu et al., 2019). The prevalence of antimicrobial resistance varies between different countries and regions (Kang et al., 2017; Jiang et al., 2019; Zhou et al., 2022). In the present study, the complete genome of multidrug-resistant *V. parahaemolyticus* strain NJIFDCVp52 from seafood was obtained by both the Illumina NovaSeq 6000 platform and the PacBio Sequel II platform. Two circular plasmids designated pVP52-1 and pVP52-2 were found in this strain, with sizes of 172,213 and 85,030 bp, respectively. pVP52-1 was classified as a IncA/C_2_-pST3 plasmid using PlasmidFinder 2.0 and pMLST, while the pVP52-2 was untypeable, which was presumably a result of continuous rearrangement and mutational events in this plasmid (Rozwandowicz et al., 2018). IncA/C is a group of low-copy number, self-transferable, broad-host-range plasmids, generally ranging in size from 40 to 230 kbp (Rozwandowicz et al., 2018; Pan et al., 2021). This incompatibility group contained two variants with 26 SNPs leading to three amino acid substitutions: IncA/C_1_ (IncA type, corresponding to the pRA1 reference plasmid) and IncA/C_2_ (IncC type), both of which were merged into the same group IncA/C (Carattoli et al., 2006; Fricke et al., 2009). The 172,213 bp MDR gene-containing plasmid pVP52-1 exhibited the highest similarity (100% coverage and 99.99% identity) to the known 163,850 bp plasmid pVb1796 (accession number: MH113855.1) from a *V. alginolyticus* strain. Similar plasmids with minor differences were also detected in *Aeromonas hydrophila*, *E. coli*, *Klebsiella pneumoniae*, and *Salmonella* strains. Furthermore, plasmid typing indicated that these plasmids belonged to the incompatibility group IncA/C_2_, and it is speculated that they may also have potential transferability. However, no highly similar plasmids were found in *V. parahaemolyticus* by BLAST service at NCBI.

The plasmid backbone of pVP52-1 is comprised of a replication region (*repA*), a stability region (*parA*, *parB*) and a conjugative transfer region (such as *traF*, *traH*, *traG*, *traI*, *traK*, *traL*). Together, these genes are involved in plasmid housekeeping functions, indicating a relatively conserved structure (Partridge et al., 2018; Rozwandowicz et al., 2018). Multiple MGEs were identified on pVP52-1, including full-length insertion sequences IS26, IS5075, IS6100, ISVsa3, etc. Noticeably, pVP52-1 harbored most of the ARGs (12/15), and pVP52-2 did not carry any ARGs. The array of resistance genes *aac(6′)-Ib*-cr-*qnrVC1*-*aac(6′)-Ib*-*dfrA14*-*mphA* was bounded by IS5075 and IS4321 in inverse orientation, suggesting an IS110-mediated composite transposon (22,778 bp), simultaneously, where there was a common IS26-*mphA*-IS6100 unit. Concomitant carriage of 5 macrolide resistance genes with different functions [*mphR*, *mrx*, *mph(K)*, *mel*, *mph2*] was reported to confer high azithromycin resistance on IncA/C plasmids from *Vibrio cholerae* serogroup O1 and O139 strains in China (Wang et al., 2018). Upstream of this array was a *dfrA1*-*bla*_CARB-4_ unit and an antimicrobial resistance module ISVsa3-*floR*-*tetA*-*aph(6)-Id*-*aph(3″)-Ib*-*sul2*, which was in line with the *V. cholerae* plasmids pVC211 and pGX0618 (Wang et al., 2018). The alignment results of variable regions emphasized that the sequential acquisition of novel resistance genes by plasmids through MGEs such as ISVsa3 and IS110-composite transposon along with a complex genetic environment might be important for the spread of diverse antimicrobial resistance profiles.

In the plasmid elimination experiment, it was found that SDS solution of a certain concentration could effectively revert the multidrug-resistant strain into a susceptible phenotype because it successfully eliminated plasmid pVP52-1, which was similar to that seen with others (Tang et al., 2018; Paul et al., 2020). On the other hand, the IncA/C_2_-type MDR plasmid pVP52-1 could be conjugatively transferred from *V. parahaemolyticus* strain NJIFDCVp52 into *E. coli* strain EC600, whereas the other plasmid pVP52-2 was not self-transmissible. It was demonstrated that transconjugant Vp52-EC600 received all 12 ARGs and gained the capability to express a multidrug-resistant phenotype accordingly. We further investigated the transferability of plasmid pVP52-1 from *V. parahaemolyticus* NJIFDCVp52 to other bacterial species, and found that pVP52-1 could be transferred to *E. coli* and *Salmonella* isolates but not to *B. cereus*, *L. monocytogenes*, *S. aureus*, and *P. aeruginosa* isolates, which may be closely related to chromosomal factors and genetic relationships between populations. Currently, the transfer characteristics of IncA/C plasmid from *V. parahaemolyticus* have not yet been reported. Some studies indicated that IncA/C plasmids are widely distributed among *E. coli*, *Salmonella* and other enterobacterial isolates (Fricke et al., 2009; Call et al., 2010), which is consistent with the findings of this study. More specifically, inconsistent results were found in resistance profiles of the wild-type strain NJIFDCVp52 and transconjugant Vp52-EC600. That is, Vp52-EC600 was more resistant to CIP, CHL, TET, and AZI. This may be because the resistance genes (*qnrVC1*, *floR*, *tetA*, and *mphA*) were better expressed in Vp52-EC600. Similar experimental phenomena have been reported in the literature (Ye et al., 2016). IncA/C plasmid pVAS3-1 in *V. alginolyticus* strain VAS3-1 could be transferred to *E. coli* J53, resulting in the changes of drug resistance (TET and CHL), but minimum inhibitory concentration values between them were different. The above results indicated that the same resistance gene may not behave consistently in different bacteria species. Liu et al. (2013) reported a novel conjugative plasmid from a *V. parahaemolyticus* strain isolated in Hong Kong with multiple ARGs, such as *bla*_PER-1_, *qnrVC6*, *aacA3*, *catB2*, *dfrA1* and *aadA1*, which conferred resistance to third-generation cephalosporins and quinolones. These data together suggested that even a single plasmid may accumulate enough ARGs to transmit antimicrobial resistance among various bacteria, highlighting the potential cumulative effects of different ARGs.

In conclusion, this study identified a conjugative IncA/C_2_-pST3 plasmid from a multidrug-resistant *V. parahaemolyticus* strain of seafood origin that carried 12 different ARGs. ISVsa3 and IS110-composite transposon were recognized as the main routes for acquiring multiple ARGs, suggesting co-transfer potential. The MDR plasmid with high stability could be transferred to enterobacterial species (*E. coli* and *Salmonella*), which played a critical role in conferring multidrug resistance. These findings emphasize the urgent need for continued surveillance of resistance plasmids and changes in antimicrobial resistance profiles among the *V. parahaemolyticus* population.

## Data availability statement

The datasets presented in this study can be found in online repositories. The names of the repository/repositories and accession number(s) can be found in the article/Supplementary material.

## Author contributions

HZ: Conceptualization, Data curation, Methodology, Writing – original draft. ZL: Conceptualization, Methodology, Writing – review & editing. XL: Methodology, Resources, Writing – review & editing. XB: Supervision, Writing – review & editing. XC: Validation, Writing – review & editing. ZW: Validation, Writing – review & editing. XS: Data curation, Writing – review & editing. JY: Resources, Supervision, Writing – review & editing.
