# Supplementary material for: Characterization and transmission of plasmid-mediated multidrug resistance in foodborne Vibrio parahaemolyticus
Source: Front Microbiol. 2024 Jul 31;15:1437660. doi: 10.3389/fmicb.2024.1437660 (PMC11322368; doi:10.3389/fmicb.2024.1437660)
Supplement: Supplementary file 1 [file Table_1.DOCX]

Supplementary Material

## Supplementary Figures


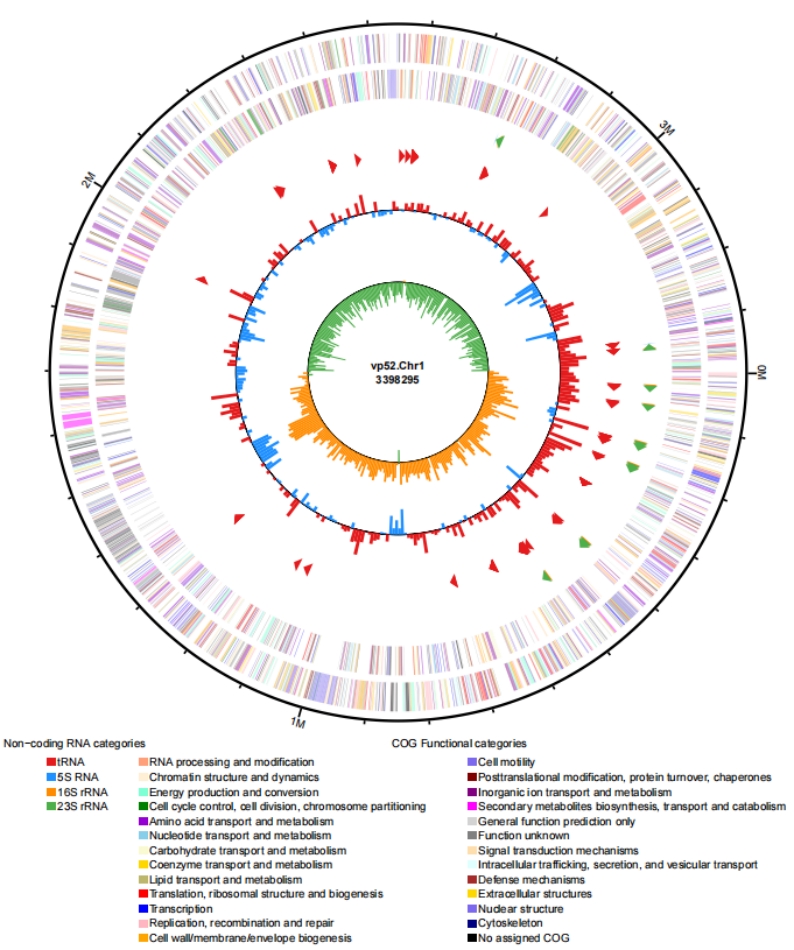


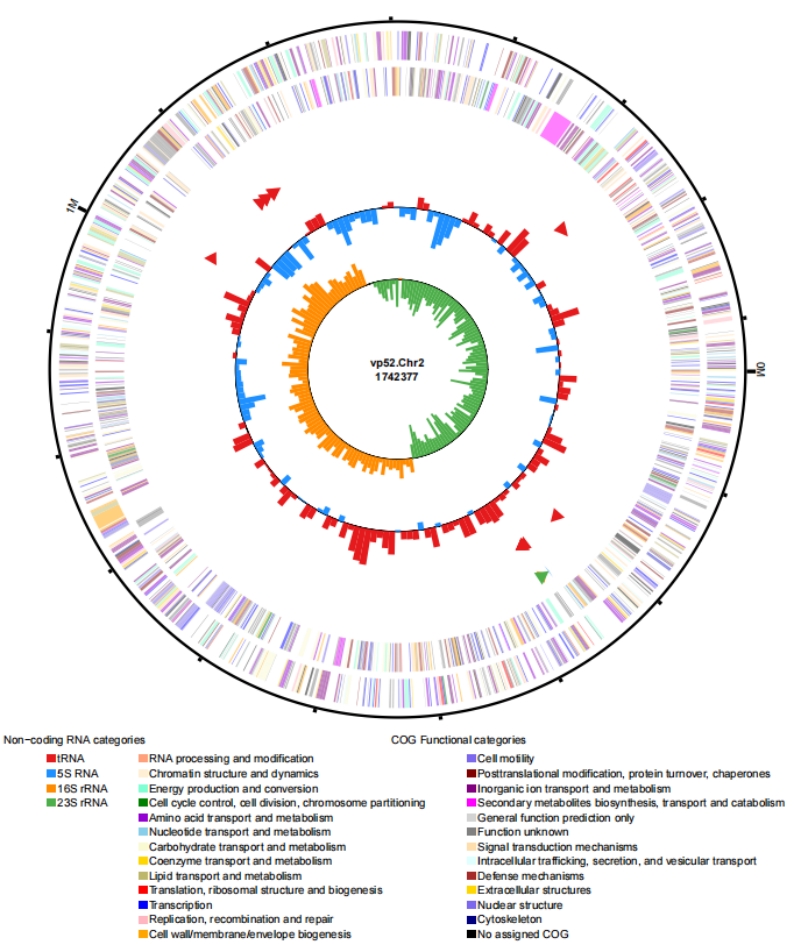


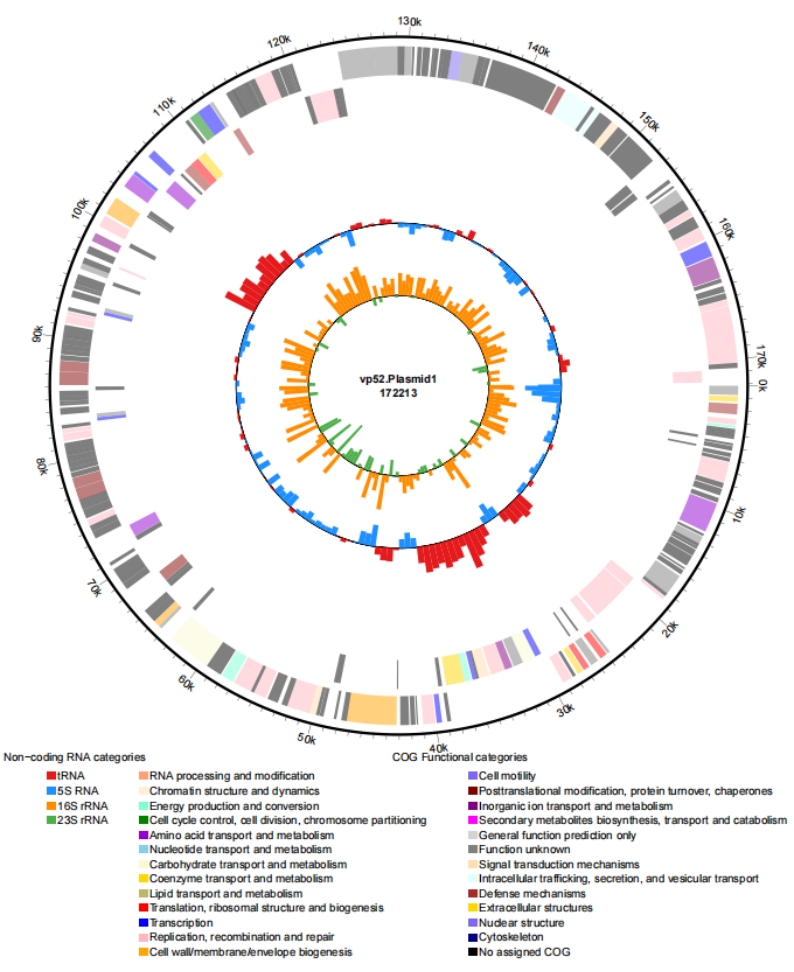


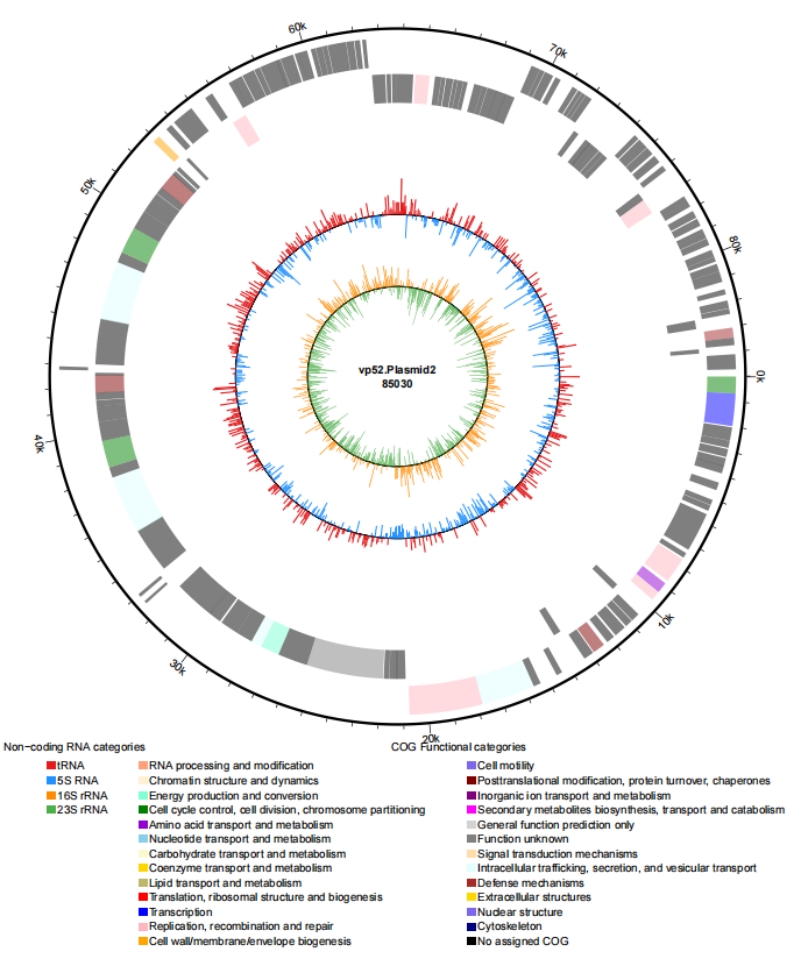


**Supplementary Figure S1.** The genome circle maps of *V. parahaemolyticus* strain NJIFDCVp52 (two chromosomes and two plasmids). From the outside inwards: the outermost circle shows the genome size; the second and third circles show the coding sequences (CDSs) on the forward and reverse strands, and different colours represent different clusters of orthologous groups (COGs) functional classifications of the CDSs; the fourth circle shows rRNA and tRNA; the fifth circle shows that the GC content of this region is higher (red) and lower (blue) than the average GC content of the whole genome; the innermost circle shows positive (yellow) and negative (green) GC skew.
