# Supplementary material for: Characterization and transmission of plasmid-mediated multidrug resistance in foodborne Vibrio parahaemolyticus
Source: Front Microbiol. 2024 Jul 31;15:1437660. doi: 10.3389/fmicb.2024.1437660 (PMC11322368; doi:10.3389/fmicb.2024.1437660)
Supplement: Supplementary file 2 [file Table_2.DOCX]

Supplementary Material

## Supplementary Tables

| Isolates | Antimicrobial agents | Minimum inhibitory concentrations (μg/mL) | Media | Isolates | Antimicrobial agents | Minimum inhibitory concentrations (μg/mL) | Media |
| --- | --- | --- | --- | --- | --- | --- | --- |
| ***Bacillus cereus*** | | | | | | | |
| BC1 | TET | 4 | MYP agar (Beijing Land Bridge, China) | BC11 | TET | 1 | MYP agar (Beijing Land Bridge, China) |
| BC2 | TET | 4 |  | BC12 | TET | 1 |  |
| BC3 | TET | 4 |  | BC13 | TET | 1 |  |
| BC4 | TET | 4 |  | BC14 | TET | 2 |  |
| BC5 | TET | 2 |  | BC15 | TET | 4 |  |
| BC6 | TET | 4 |  | BC16 | TET | 4 |  |
| BC7 | TET | 1 |  | BC17 | TET | 2 |  |
| BC8 | TET | 1 |  | BC18 | TET | 4 |  |
| BC9 | TET | 1 |  | BC19 | TET | 4 |  |
| BC10 | TET | 2 |  | BC20 | TET | 4 |  |
| ***Salmonella*** | | | | | | | |
| *S*. Indiana SC109 | TET | 2 | Salmonella chromogenic agar (HuanKai Microbial, Guangdong, China) | *S*. Indiana SC124 | TET | 4 | Salmonella chromogenic agar (HuanKai Microbial, Guangdong, China) |
| *S*. Derby  SC110 | TET | 4 |  | *S*. Typhimurium SC125 | TET | 2 |  |
| *S*. Chester SC111 | TET | 2 |  | *S*. Indiana SC126 | TET | 1 |  |
| *S*. Derby  SC112 | TET | 4 |  | *S*. Indiana SC157 | CTX | ≤0.25 |  |
| *S*. Typhimurium SC113 | TET | 4 |  | *S*. Typhimurium SC158 | CTX | ≤0.25 |  |
| *S*. Typhimurium SC114 | TET | 2 |  | *S*. Indiana SC159 | CTX | ≤0.25 |  |
| *S*. Chester SC115 | TET | 4 |  | *S*. Derby  SC160 | CTX | 2 |  |
| *S*. Indiana SC116 | TET | 1 |  | *S*. Typhimurium SC170 | CTX | 4 |  |
| *S*. Indiana SC122 | TET | 2 |  | *S*. Indiana SC173 | CTX | 4 |  |
| *S*. Typhimurium SC123 | TET | 4 |  | *S*. Typhimurium SC174 | CTX | ≤0.25 |  |
| ***Escherichia coli*** | | | | | | | |
| EC1 | CTX | ≤0.25 | EMB agar (Beijing Land Bridge, China) | EC26 | CTX | ≤0.25 | EMB agar (Beijing Land Bridge, China) |
| EC2 | CTX | ≤0.25 |  | EC27 | CTX | 4 |  |
| EC3 | CTX | ≤0.25 |  | EC28 | CTX | ≤0.25 |  |
| EC4 | CTX | ≤0.25 |  | EC29 | CTX | ≤0.25 |  |
| EC5 | CTX | 1 |  | EC30 | CTX | ≤0.25 |  |
| EC6 | CTX | 1 |  | EC31 | CTX | 1 |  |
| EC7 | CTX | ≤0.25 |  | EC32 | CTX | 1 |  |
| EC8 | CTX | ≤0.25 |  | EC33 | CTX | ≤0.25 |  |
| EC9 | CTX | 1 |  | EC34 | CTX | ≤0.25 |  |
| EC10 | CTX | ≤0.25 |  | EC39 | CTX | 1 |  |
| ***Pseudomonas aeruginosa*** | | | | | | | |
| PA1 | CAZ | ≤1 | Pseudomonas CN selective (Beijing Land Bridge, China) | PA6 | CAZ | ≤1 | Pseudomonas CN selective (Beijing Land Bridge, China) |
| PA2 | CAZ | 2 |  | PA7 | CAZ | ≤1 |  |
| PA3 | CAZ | 2 |  | PA8 | CAZ | 2 |  |
| PA4 | CAZ | ≤1 |  | PA9 | CAZ | ≤1 |  |
| PA5 | CAZ | ≤1 |  |  |  |  |  |
| ***Listeria monocytogenes*** | | | | | | | |
| LM1 | TET | 1 | ALOA agar (Beijing Land Bridge, China) | LM5 | TET | 2 | ALOA agar (Beijing Land Bridge, China) |
| LM2 | TET | ≤0.5 |  | LM6 | TET | 1 |  |
| LM3 | TET | 1 |  | LM7 | TET | 1 |  |
| LM4 | TET | ≤0.5 |  |  |  |  |  |
| ***Staphylococcus aureus*** | | | | | | | |
| SA1 | TET | 2 | Baird-Parker agar (Beijing Land Bridge, China) | SA11 | TET | 4 | Baird-Parker agar (Beijing Land Bridge, China) |
| SA2 | TET | 1 |  | SA12 | TET | ≤0.5 |  |
| SA3 | TET | ≤0.5 |  | SA13 | TET | 1 |  |
| SA4 | TET | ≤0.5 |  | SA14 | TET | ≤0.5 |  |
| SA5 | TET | ≤0.5 |  | SA15 | TET | 4 |  |
| SA6 | TET | 2 |  | SA16 | TET | ≤0.5 |  |
| SA7 | TET | 4 |  | SA17 | TET | ≤0.5 |  |
| SA8 | TET | 2 |  | SA18 | TET | ≤0.5 |  |
| SA9 | TET | ≤0.5 |  | SA19 | TET | ≤0.5 |  |
| SA10 | TET | ≤0.5 |  | SA20 | TET | ≤0.5 |  |

TET, tetracycline; CTX, cefotaxime; CAZ, ceftazidime.

**Supplementary Table S1. Strain information used for conjugation transfer.**
